# Collective order and group structure of shoaling fish subject to differing risk-level treatments with a sympatric predator

**DOI:** 10.1098/rsos.231511

**Published:** 2024-05-15

**Authors:** Timothy M. Schaerf, Alexander D. M. Wilson, Mitchell Welch, Ashley J. W. Ward

**Affiliations:** ^1^ School of Science and Technology, University of New England, Armidale, New South Wales, Australia; ^2^ School of Life and Environmental Sciences, University of Sydney, Sydney, New South Wales, Australia; ^3^ School of Biological and Marine Sciences, University of Plymouth, Devon PL4 8AA, UK

**Keywords:** predator–prey, threat, collective movement, group order, *Gambusia holbrooki*, rules of interaction

## Abstract

It is imperative for individuals to exhibit flexible behaviour according to ecological context, such as available resources or predation threat. Manipulative studies on responses to threat often focus on behaviour in the presence of a single indicator for the potential of predation, whereas in the wild perception of threat will probably be more nuanced. Here, we examine the collective behaviour of eastern mosquitofish (*Gambusia holbrooki*) subject to five differing threat scenarios relating to the presence and hunger state of a jade perch (*Scortum barcoo*). Across threat scenarios, groups exhibit unique behavioural profiles that differ in the durations that particular collective states are maintained, the probability of transitions between states, the size and duration of persistence of spatially defined subgroups, and the patterns of collective order of these subgroups. Under the greatest level of threat, subgroups of consistent membership persist for longer durations. Group-level behaviours, and their differences, are interconnected with differences in estimates of the underlying rules of interaction thought to govern collective motion. The responses of the group are shown to be specific to the details of a potential threat, rather than a binary response to the presence or absence of some form of threat.

## 1. Introduction

Grouping with others is an integral part of the lives of many species [1]. At a practical level, there are advantages to grouping that may include sharing of information and resources [2], collective vigilance [3], reduced probability of being a victim of predation when an attack does occur (via attack abatement and confusion effects) [4–7] and an enhanced ability to explore and navigate the environment [8].

Many social and gregarious species engage in a range of collective behaviours, including tasks that involve collective decision making [9] and coordinated movement [1]. Decision-making tasks may include choices relating to foraging [10], the identification of a viable new home for the group [2] and the decision for a group to stay in a particular location or move on [11]. These cases may all then lead to the need for a group to coordinate collective motion, a phenomenon that is seen repeatedly throughout nature [1], across a range of scales and species, from the movements of cells [12] to truly vast assemblages such as krill swarms [13,14].

In many cases, the patterns of collective motion are thought to emerge due to repeated application of local ‘rules of interactions’ that govern how individuals adjust their velocity, and hence their trajectory, as a function of the relative positions and behaviours of neighbouring members of a group. A broad range of theoretical studies have shown that emergent patterns of motion that visually resemble those seen in nature, such as swarming, milling, directed parallel motion and a variety of more complex patterns, can be generated via models that employ basic, locally defined rules of interaction [15–24]. In most cases, these social interaction rules include at least one of the following mechanisms: (i) a rule for collision avoidance, especially via repulsion from near neighbours; (ii) a rule for orienting each individual’s direction of motion with that of neighbours; and (iii) an explicit rule for maintaining group cohesion, via attraction to neighbours or the group centre. Further, a number of studies have shown systematically that adjusting the details of how these interactions apply, for example, by modifying the spatial range over which each of the three rules above operates, can affect the emergent patterns of motion [20,23,24]. More recent work has begun to identify the presence and form of rules of interaction, like those hypothesized for model construction, directly from tracking data via techniques that include analysis of local group structure [25], comparison of local alignment and structure between observations and simulation models [26], social force matching [27], methods aimed at constructing data-driven models [28–32], social force mapping (a distinct technique to force matching) [31,33,34] and machine learning [35]. In total, empirical studies suggest the presence of elements of repulsion [31,33,34,36], orientation [31,37,38] and attraction [31,33,34,36] in the manner in which real individuals adjust their velocity in response to neighbours in moving groups. The rules of interaction inferred from data are similar to those proposed in models in broad concept, but sometimes differ with the models in detail, and species studied thus far do not necessarily exhibit all three of repulsion-, orientation- and attraction-like behaviour in concert.

A central tenet of studies of collective motion, whether theoretical or empirical, has been the quantification of the structure of moving groups, through the spacing, relative positioning and alignment of individuals [25,26], and the degree of order of the pattern of movement, through collective order parameters [20,23,39]. One of the most commonly applied order parameters is polarization, which measures the instantaneous alignment between all group members [20,23]; high values of this parameter are often associated with coordinated, directed parallel motion by all group members. Measures have also been developed to quantify how close the movement of a group is to perfect milling motion about a central point in the group, with one of the most commonly applied being the group angular momentum [23,39]. Combined, polarization and angular momentum parameters can be used to identify some of the fundamental patterns of collective motion, especially swarming, milling and parallel motion [23,39–41].

Beyond a general interest in classifying the broad patterns of collective motion, collective order analysis has a connection with assessing the ability of some species to avoid predation, and the responses of some species as a collective to varying predation risk. For example, predators may become less able to identify individual target prey when the behaviour and appearance of potential prey is more uniform (the ‘confusion effect’ [1]). Experimental work has demonstrated that the rate at which three-spined sticklebacks (*Gasterosteus aculeatus*) attack swarms of *Daphnia magna* declines when individual *Daphnia* move parallel or perpendicular to each other [42]. When exposed to increased predation risk, some species have been observed to adjust their alignment with other group members, including reducing, rather than increasing polarization [36]. Additional responses to predation risk include reducing distances to near neighbours, reducing speed and general activity, increasing vigilance behaviour and increasing the use of refuges [36,43–45].

Some prior studies on the effects of predation risk on individual and collective behaviour have involved the presence or absence of a consistent indicator of predation risk. Hoare *et al*. [46] examined the effects of food and alarm cues on the formation of groups in shoals of 10 banded killifish (*Fundulus diaphanous*). It was found that shoals of killifish subject to an alarm cue, or a combination of food and alarm cues, tended to form groups with a greater median group size than control shoals; this is consistent with individuals positioning themselves more closely to neighbours in a situation of perceived risk. Schaerf *et al*. [36] conducted a wide-ranging analysis of the behaviour of shoals of eight X-ray tetras (*Pristella maxilliaris*), subject to either food or alarm cues, along with control observations, but no combination of both food and alarm cues. Alarm cues in particular had a pronounced effect on the behaviour of the X-ray tetras across multiple measures compared with control treatment shoals, including reduced speed of individuals, increased turning speed and frequency of rapid turns, reduced magnitude of acceleration, reduced neighbour distances, increased statistical density of near neighbours (somewhat equivalent to increased group size for killifish observed in [46]), and reduced polarization and maximum alignment when aligning with neighbours. There was also evidence that alarm cues affected the underlying rules of interaction applied by the X-ray tetras, particularly those connected to collision avoidance/repulsion and cohesion/attraction. It appeared that the size of the region over which repulsion applied may have reduced, evidenced by a reduction in the size of an apparent avoidance zone in the statistical density of neighbours and a muting of speed-mediated collision avoidance behaviour at close range. In addition, the X-ray tetras tended to turn more rapidly towards neighbours when alarm cues were present.

Given the complexity of the natural environment, it is likely that real animals must adjust their behaviour in response to a spectrum of ecological conditions, including risk scenarios, rather than the simple presence or absence of cues indicating the presence of food or a recent instance of predation, or a simple combination of these two forms of cue. Recent work in [47] has sought to extend understanding of experimentally manipulated responses to predation threat beyond the binary presence or absence of alarm cues. Groups of eight females eastern mosquitofish (*Gambusia holbrooki*) were subject to five different scenarios designed to mimic differing potential or perceivable levels of threat. The differing threat levels involved the presence or absence of a sympatric predator (a jade perch, *Scortum barcoo*) or its chemical cues, where the predator was subject to differing feeding regimes prior to observations. The structure of the experimental arena played a key role in behavioural observations, and comprised a central zone with relatively deep water that sometimes contained the predator, bounded by two platform regions with much shallower water. Analysis of the movement behaviour of the mosquitofish in [47] largely focused on individual measures, or those focused around individuals. Unsurprisingly, the mosquitofish preferred the platform regions of the tank when the perch (predator) occupied the central zone, and the deeper water of the central zone when the predator was not present in this zone. The fish also adjusted their behaviour dependent on treatment and their occupancy of either their preferred or non-preferred zone. Crossing from platform to platform was less frequent when there were only predator cues, or the predator was unfed and present in the central zone, than in the cases that the predator had been fed, or there was no predator. Treatment, zone occupied and the interaction between these two were all associated with significant differences in individual speed, nearest neighbour distances, polarization, and mutual information and entropy rate associated with changes in position and velocity. There was also evidence that neighbour density, local alignment and interaction rules varied significantly between many pairs of treatments.

Here, we extend the work presented in Wilson *et al*. [47], with a focus on aspects of the collective behaviour of the groups in that study. We examine the effect of varying risk scenario treatments on the form and persistence of collective states, quantified via collective order parameters of polarization (previously used in isolation in [47]) and group angular momentum, and the possible association between any differences in states and underlying rules of interaction (inferred via force mapping, previously reported in [47]). We also examine the formation, maintenance, structure and collective order of subgroups of varying size that arose during different treatments, with subgroup membership identified via a distance-based threshold, similar to that employed by Hoare *et al*. [46].

## 2. Methods

### 2.1. Experimental set-up

**Figure 1.**
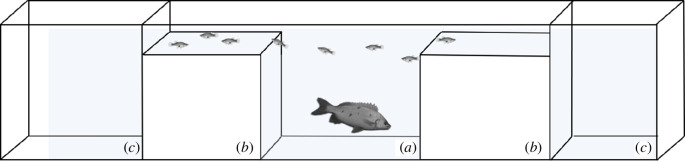
Schematic representation of experimental arena used across risk gradient treatments. The arena consisted of three primary zones as represented by a central open water zone (*a*) flanked by two shallow platform zones (*b*) on either side. When the predator (*S. barcoo*) was present visually (T3–T5) it was located in the central zone. Across treatments, mosquitofish (*G. holbrooki*) could move across the three zones (*a* and *b*) freely but were prevented from reaching the outermost areas (*c*) beyond the platforms by an opaque partition. In the predator cues treatment (T2), the predator was located in one of these two outermost areas on either side of the platform zones and water (containing cues) was mixed with the experimental observation area. The width of the tank was 30 cm, and the tank was filled to a depth of 17 cm with aged, conditioned tap water. The central zone (*a*) and each of the platform regions (*b*) were 22 cm long. The heights of the platforms were 15 cm, such that the depth of water was 2 cm above the platforms. The outermost areas (*c*) were 27 cm in length. (Adapted from fig. 1 in [47].)

The experimental subjects for this study were wild-caught female eastern mosquitofish (*Gambusia holbrooki*, total body length: 23 ± 2 mm); full details are provided in Wilson *et al*. [47] and the supplementary information for this study for completeness. Groups of eight novel fish were selected haphazardly from the population of wild-caught fish, and initially moved to the right platform of the experimental arena pictured in figure 1 (platform regions are labelled with *b*). The groups of fish were subject to one of five treatments that represented different potential levels of predation threat. The treatments were:

T1—a control treatment where the experimental aquaria was empty, other than the mosquitofish;
T2—a sympatric predator (a jade perch, *S. barcoo*, total body length: 128 mm) that had recently been fed to satiation on dead mosquitofish was placed in one of the outermost regions (figure 1*c*) 90 min prior to the release of the mosquitofish, and the water in the partitioned region was mixed with the central region, so that the presence of the predator would be evident via chemical cues;
T3—the predator (jade perch) was fed to satiation on commercial pellets for fish before being released in the central zone (figure 1*a*) 90 min prior to the release of the mosquitofish;
T4—the predator (jade perch) was fed to satiation on dead mosquitofish before being released in the central zone (figure 1*a*) 90 min prior to the release of the mosquitofish; and
T5—the predator (jade perch) was released in the central zone (figure 1*a*) without prior feeding on dead mosquitofish or pellet food; again, the predator (perch) was released in the arena 90 min prior to the release of the mosquitofish.

After their release within the right platform region, the mosquitofish were allowed to swim and explore freely for 15 min before their movements were videoed from above over a period of 20 min at a rate of 25 frames per second. Each mosquitofish was only used for one experimental trial, to eliminate bias based on prior experience with the arena, predator or experimental procedure. The same perch was used for all trials that included a predator (see [47] for further discussion). We sought to perform as many trials as possible based on available time and the number of mosquitofish available. Ultimately each treatment consisted of 10–13 replicates, with 13 replicates for T1, 10 replicates for T2, 12 replicates for T3, 11 replicates for T4, and 13 replicates for T5.

### 2.2. Tracking data

Post-experimental trials, we used the automatic visual tracking software CTrax [48] to deduce time series of each individual’s coordinates in two dimensions (when viewed from above). The two-dimensional approximation to the movements was reasonable, since the fish tended to swim at approximately the same level, near the water’s surface, throughout all trials, dictated by the height of the platforms, with little variation in the depth at which they swam. Any ambiguities or potential errors in the time series/trajectories were resolved using the *fixerrors* GUI in MATLAB. Throughout this paper, we denote the resulting inferred coordinates of individual *i* at time *t* during a given trial and as deduced via the automated tracking as $\left(x_{i}\left(t\right),\;y_{i}\left(t\right)\right)$ . Data from each experimental trial comprised eight trajectories spanning 30 000 discrete time steps (from 20 min of video footage at 25 frames per second).

### 2.3. Analysis

All analysis was performed via custom scripts developed in MATLAB (R2021a; The Mathworks, Inc., Natick, MA). We smoothed the tracked *x* and *y* coordinates of each individual using a Savitzky–Golay filter with a span of five data points and degree 2, prior to applying any of the analyses outlined below.

#### 2.3.1. Force-map estimates for the components of a change in velocity as a function of relative neighbour coordinates

We re-examined force-map estimates for the form of the components of individuals’ changes in velocity, measured via changes in speed (the magnitude of velocity) over time and change in direction of motion (heading) over time as a function of the relative position of neighbours, as were originally constructed in Wilson *et al*. [47]. The re-examination was to enable direct juxtaposition of inferred interactions, and differences in these interactions across treatments, with measured group-level patterns of movement, described further below. The maps are constructed using a consistent frame of reference, such that a ‘focal individual’ is positioned at the origin (0, 0), and the direction of motion of the focal individual is parallel to the positive *x*-axis. The maps show how an individual adjusts the components of its velocity on average when other group members occupy particular (*x*, *y*) coordinates. Details of the force-mapping calculations and some notes about the accuracy of the method are included in the electronic supplementary material. The force-map analysis applied here is identical to that in [47], but the method of presentation is different; in [47] separate force maps are presented for the behaviour of individuals in the central chasm and outer platform regions, whereas here we aggregate data to generate single maps for behaviour across the entire tank. These single maps per treatment were those used in subsequent randomization comparisons of collective motion rules of interaction across treatments; we refer to the randomization results in [47] again in this paper, and outline the randomization procedure following the description of the construction of force maps in the electronic supplementary material.

#### 2.3.2. Collective order and states of groups

We characterized the patterns of motion of entire groups via the order parameters of group polarization, $O_{p}$ , and group angular momentum, a measure of rotational order, denoted $O_{r}$ [23,39–41]. Both order parameters are measured on a scale from 0 to 1, and are derived from data at a specific time instant *t*. $O_{p}\left(t\right)\;=\;1$ indicates perfect alignment in directions of motion of individuals at a given instant, with lower values of polarization coinciding with lower levels of similarity in directions of motion. Group angular momentum characterizes the mill-like elements of group movement, with $O_{r}\left(t\right)\;=\;1$ indicative of a perfect mill about the group centroid in a single sense (either clockwise or anticlockwise), and lower values of the angular momentum suggesting lesser agreement in the sense of rotation about the group centre by individuals. Full details for the calculation of both order parameters are provided in the electronic supplementary material. We pooled all pairs of values for polarization and rotational agreement, $O_{p}\left(t\right)$ and $O_{r}\left(t\right)$ , respectively, for all observed times within each treatment. We constructed plots of the relative frequency that each pair $\left(O_{r}\left(t\right),\;O_{p}\left(t\right)\right)$ was observed, rendered as heat maps using MATLAB’s *surf* function. The plots were smoothed via overlapping bins and constructed in a manner similar to overlapping bins used in the force-mapping procedure (see the electronic supplementary material for further details). Following the classification method used by Tunstrøm *et al*. and Welch *et al*. [39,40], we classified subgroup motion where $O_{r}\left(t\right)<0.35$ and $O_{p}\left(t\right)<0.35$ as swarming (S), motion where $O_{r}\left(t\right)<0.35$ and $O_{p}\left(t\right)>0.65$ as parallel motion (P) and motion where $O_{r}\left(t\right)>0.65$ and $O_{p}\left(t\right)<0.35$ as milling (M). In [39,40], all other motion with order parameters outside the above ranges was classified as transitional, specifically referring to periods in which the group reorganized from one fundamental emergent state to another (e.g. from milling to parallel motion). Welch *et al*. [40] noted that such transitional states are composed of a mixture of behaviours, including elements of polar, swarming and milling formations. Theoretical work [41] suggests that states corresponding to this larger region of parameter space can persist for long durations, so they are not necessarily transitional, with specific examples given in Mudaliar *et al*. [41] of simulations with persistent motion with combinations of mill-like motion and directed motion, or swarm-like motion and directed motion. Here, we refer to emergent patterns coinciding with the larger region of order parameter space as composite motion (C), given that such motions are probably compositions of swarming, milling and parallel motion. In addition to direct analysis of order parameters, we examined the mean direction of change of collective order parameters through $\left(O_{r},\;O_{p}\right)$ space; at a given instant we denoted this direction via ψ(t) (see the electronic supplementary material for full details of these calculations).

We tallied the number of frames in each experimental replicate that groups were in each of the milling, parallel, swarming and composite states, and from these tallies determined the proportion of the observation time spent in each state. For visualization purposes, we constructed box plots illustrating the proportions of time spent in each state (one set of box plots per state), along with a graph illustrating the proportion of all time (across all replicates within a given treatment) spent in each state. We sought to compare the proportions of time spent in each state across treatments; to do this we tested the potential normality of each set of proportions for each treatment using either Shapiro–Wilk tests [49] or Shapiro–Francia tests [50] as appropriate. In addition, we examined the equality of variances via Levene’s test computed by performing ANOVA on the absolute deviations of the data values from the set means. In most, but not all, instances, the data were consistent with normality, but variances were not equal for the milling, parallel and swarming states, and the control set was not consistent with normality for the composite state. We thus applied a bootstrapping approach to make pairwise comparisons between treatments of the proportions of time spent in each state, with basic bootstrap confidence intervals constructed following the theory in [51]. The bootstrap scheme that we applied is detailed in the electronic supplementary material.

We identified the durations that groups maintained particular states throughout each experimental replicate. We applied standard techniques of survival analysis, as described in Kalbfleisch & Prentice [52], to determine if different collective states persisted for different durations within the same treatment, or if like states differed in duration of persistence across treatments. If a collective state was active at the first or last tracked frame for a given trial, then we treated the duration of persistence for that particular state as right censored (where the duration of persistence of the state was at least that observed), and all other durations were treated as uncensored. We pooled the sets of censored and uncensored durations for which given states (milling, parallel motion, swarming or composite motion) persisted from data for all trials within each treatment. For visualization, and to aid in interpreting our analysis, we constructed Kaplan–Meier estimates of the survival functions associated with the duration that each state persisted, along with approximate bounds for the 95% confidence interval for each survival function. The survival functions, S(t) , represented the probability that a collective state persisted for more than t seconds. Within each treatment, or for each state, we performed a log-rank test to determine the probability that at least one survival function differed from the others (using the procedure described in [52]). If there was a significant difference (at significance level 0.05), we then performed pairwise comparisons of all possible pairs of survival functions using additional log-rank tests to determine which survival curves in fact differed. We sorted the results of all pairwise comparisons in ascending order of *p*-value (and descending order of test statistic), and identified significant differences in pairwise comparisons after applying a Holm–Bonferroni correction [53]. We then used the Kaplan–Meier estimates of the survival functions to identify which states tended to persist for shorter or longer durations than others.

Finally, we examined the transitions between collective states for whole groups, by determining the relative frequencies that groups transitioned between the states from one video frame to the next, aggregating data across all experimental trials within each particular treatment.

#### 2.3.3. Formation, persistence and average structure of subgroups

We used the algorithm described by Hansen *et al*. [54,55] to classify fish as being part of subgroups based on the distances between individual fish. We identified distinct subgroups as sets of mosquitofish where no member was more than four body lengths (100 mm) from any other fish within the group. The four body-length criterion is within the range of three to five body lengths that has been used in previous studies of shoaling fish to identify group members based on distance to other fish [46,54–57]. We determined the durations that each subgroup persisted, with each duration ending when the combination of fish that defined the subgroup was no longer together. There was no grace period applied for the continued persistence of subgroups, so, for example, if an individual moved outside the threshold distance of 100 mm from all other subgroup members for any duration from one video frame and above, then this departure from the subgroup would have concluded that particular period of persistence for the subgroup, even if the individual rejoined the subgroup shortly thereafter. Similar to the analysis of the persistence of collective states for the whole group, we applied survival analysis via log-rank tests and Kaplan–Meier estimates for survival functions with 95% confidence intervals to determine if subgroups of different sizes persisted for different durations within the same treatment, or if subgroups of the same size differed in duration of persistence across treatments. If a subgroup was present at the first or last tracked frame for a given trial, then we treated the duration of persistence for that particular grouping as right censored (where the duration of the grouping was at least that observed), and all other durations were treated as uncensored. We pooled the sets of censored and uncensored durations for which groups of sizes one to eight persisted from data for all trials within each treatment.

**Figure 2.**
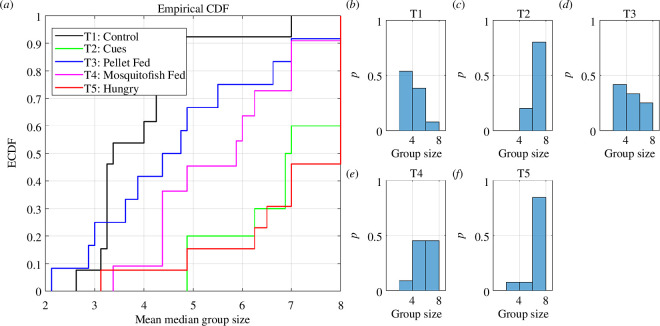
Empirical cumulative density functions (*a*) and relative frequency histograms (*b–f*) of the mean median subgroup size observed for each treatment. Each curve in (*a*) shows the probability of the mean median subgroup size occupied by individuals being less than or equal to that on the horizontal axis for T1 (control—black), T2 (predator cue—green), T3 (predator fed with pellets—blue), T4 (predator fed with mosquitofish—magenta) and T5 (hungry predator—red) treatments. For the histograms (*b–f*), the number of trials was as follows: (T1) control, *n* = 13; (T2) predator cues, *n* = 10; (T3) pellet-fed predator, *n* = 12; (T4) mosquitofish-fed predator, *n* = 11; and (T5) hungry predator, *n* = 13.

We also constructed time series of the size of the subgroup occupied by each fish for each video frame. We determined the median across all tracked frames of the subgroup size occupied by each fish, and then determined the mean across fish of the median subgroup size occupied by each fish as a summary statistic of grouping behaviour within each trial. Similar to the analysis of proportions of time spent in particular states, in many cases, distributions of mean median subgroup size differed significantly from normality (according to Shapiro–Wilk and Shapiro–Francia tests), and inspection of histograms suggested that the overall shapes of the distributions also differed between treatments (see figure 2 *b*–*f*). We thus performed a series of pairwise comparisons between treatments to determine if there were significant differences in distributions of mean median subgroup size using two-sample Kolmogorov–Smirnov tests [58–60], as implemented by MATLAB’s *kstest2* function. To account for the multiple pairwise comparisons between treatments (10 pairs in total), we again applied Holm–Bonferroni method [53] to adjust significance levels. In the case that there were significant differences between treatments, we then identified the general nature of this difference by examining graphs of the empirical cumulative density function for mean median subgroup sizes occupied by individuals for each treatment. The cumulative density functions here give the probability of individuals having mean median subgroup sizes less than or equal to a specific value.

We examined the average structure of subgroups of two to eight members within each treatment via plots illustrating the relative frequency that subgroup members occupied given positions relative to the position and direction of motion of the subgroup centroid, and the relative alignment of individuals with the direction of motion of the group centroid when in given positions. The construction of these plots is similar to that of force-map estimates for changes in speed or direction, with the primary difference being that the position and velocity of each subgroup’s centre was used as a reference point, rather than the position and velocity of individual group members. Further details of the calculations are provided in the electronic supplementary material.

#### 2.3.4. Patterns of subgroup movement in different zones

We examined the patterns of movement of subgroups of two to eight members (as classified using the algorithm from Hansen *et al*. [54,55]) when all subgroup members were either fully contained within the central region, or one of the platform regions, via the polarization of subgroup members and angular momentum, using the same form of calculations described above for the whole group. Data where all subgroup members were not in the same region of the tank were excluded from this analysis. Following Wilson *et al*. [47], we examined these data in terms of the form of the region where individuals spent most of their time during observations. For T1 and T2 trials, the region where individuals *preferred* to spend their time/spent the majority of their time was the central chasm region (*a* in figure 1, and refer to [47]). Individuals in T3–T5 treatments preferred the shallower and more protected water of the platform regions (*b* in figure 1, and refer to [47]). The *non-preferred* areas of the tank were the platforms for T1 and T2, and the central chasm region for T3–T5 (all determined by prior analysis in [47]).

## 3. Results

### 3.1. Force-map estimates for repulsion and attraction interactions

**Figure 3.**
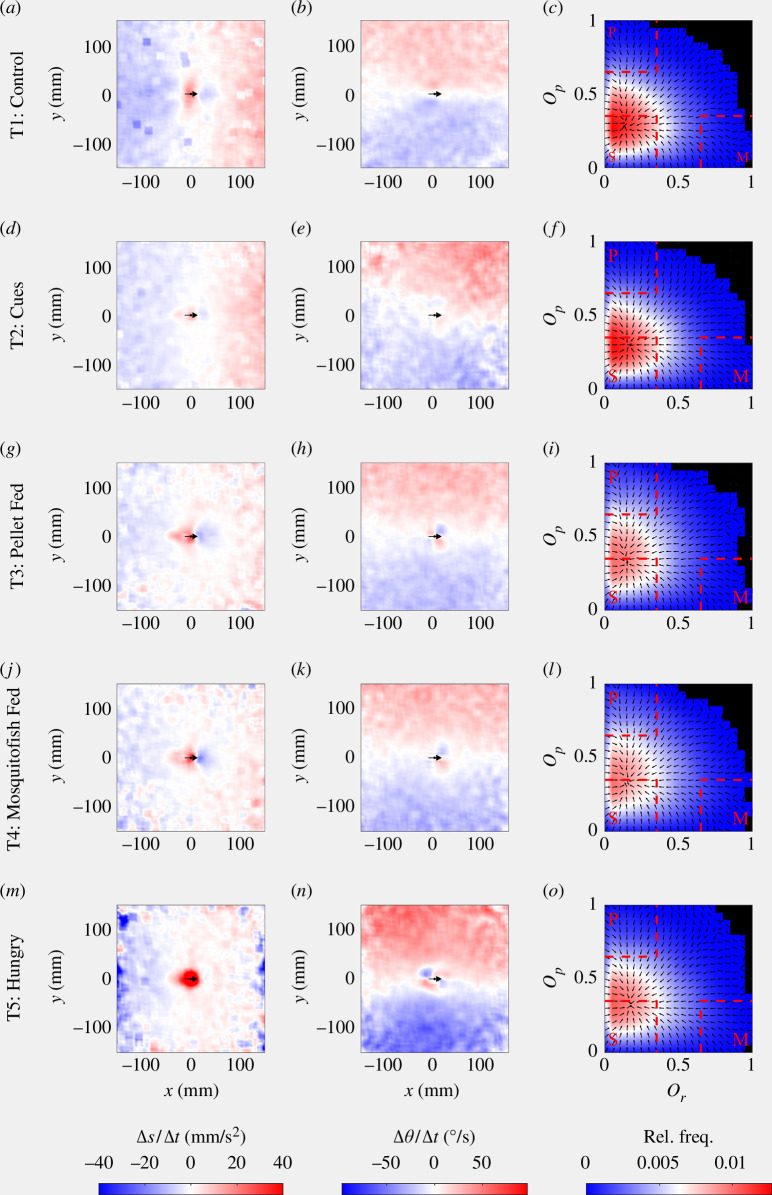
Force-map estimates for individual changes in speed (*a*, *d*, *g*, *j*, *m*) and direction of motion (*b*, *e*, *h*, *k*, *n*) as a function of the relative coordinates of neighbouring fish, and the relative frequencies of collective order parameter pairs (*c*, *f*, *i*, *l*, *o*). Treatments increase from T1 to T5 moving down the rows in the above plots. In the left and middle columns, the focal individual is located at the origin (0, 0), with its direction of motion parallel to the positive *x*-axis (travelling from left to right). The position and direction of motion of the focal individual is illustrated by a black arrow in the leftmost and middle columns. Positive changes in speed are rendered in red, and negative changes in speed in blue in the left column; the colour scale for the change in speed plots has been truncated at ±40 mm s^−2^, such that the darkest colours indicate speed changes of at least 40 mm s^−2^ in magnitude. Positive changes in direction of motion (corresponding to anticlockwise or left turns by the focal individual) are rendered in red, and negative changes in direction (corresponding to clockwise or right turns) are rendered in blue in the middle column. In the relative frequency plots in the right column, regions in parameter spacing corresponding to milling (M), parallel motion (P) and swarming (S) are demarcated by red dashed lines. Any $\left(O_{r},\;O_{p}\right)$ pairs outside the milling, parallel motion and swarming regions were treated as being representative of composite motion. Black regions in the plots outside the main coloured regions indicate zero relative frequency. Arrows superimposed on the relative frequency plots indicate the mean direction of change in parameter space (an estimate for the direction of $dO_{p}/dO_{r}$ ).

**Figure 4.**
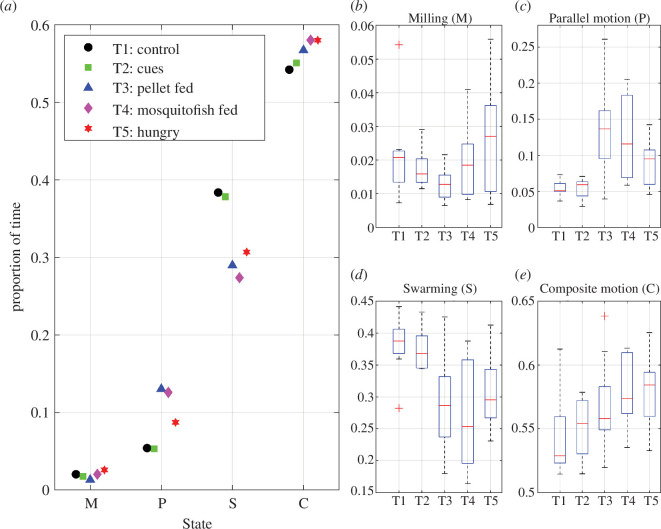
Total proportions of time spent in collective states automatically classified as milling (M), parallel motion (P), swarming (S) or composite motion (*c*). (*a*) Illustrates the total proportion of time spent in each state using data aggregated across all observations within each treatment. The boxplots, (*b*) to (*e*), were derived from the proportion of time spent in each state for each experimental trial.

As originally reported in Wilson *et al*. [47], there were significant differences between multiple pairs of states in how individuals adjusted their speed and direction of motion as a function of the relative coordinates of neighbours as inferred via force mapping (electronic supplementary material, tables S1 and S2, mean absolute difference randomization tests). There were significant differences in both components of a change in velocity (change in speed and direction of motion) between T1 (control) and T4 (mosquitofish-fed predator), T1 and T5 (hungry predator), and T3 (pellet-fed predator) and T5 treatments. T1 and T3, T2 (predator cue) and T3, and T2 and T4 treatments differed only in how individuals adjusted their speed as a function of the relative coordinates of their neighbours. Differences in how individuals adjusted their direction of motion only were inferred between T2 and T5, and T4 and T5 treatments. There were no differences in how individuals adjusted their direction of motion between T1 and T2 treatments, where there was no predator in the central zone, and T3 and T4 treatments, where there was a satiated predator in the central zone. The qualitative form of the inferred interactions for the T1 through T4 treatments was similar to that of previous studies of eastern mosquitofish [34,61]. The fish reduced their speed when neighbours were within 40 mm to their front, and increased their speed when neighbours were within 40 mm to their rear, consistent with the moderation of speed to avoid collisions (figure 3, leftmost column; compare with figs. 1A and 1B and the top row of fig. S5 in [34] and the top rows of figs. 4 and 6 in [61]). When neighbours were outside the collision avoidance zone, but to the front of the focal fish, then the focal fish would increase their speed, and when neighbours were to the rear at these intermediate distances, then the focal fish would reduce their speed, consistent with moderation of speed to avoid spatial separation from neighbours (figure 3, leftmost column; again, compare with figs. 1A and 1B and the top row of fig. S5 in [34] and the top rows of figs. 4 and 6 in [61]). In general, in the T1–T4 treatments, the mosquitofish would turn towards neighbours at distances greater than approximately 20 mm (figure 3, middle column; compare with figs. 1E and 1F and the bottom row of fig. S5 in [34], and figs. 8 and 9 in [61]). In addition, fish in the T3 and T4 treatments exhibited a tendency to turn away from very near neighbours to their front, and away from very near neighbours to their rear (figure 3, middle column; and as previously reported for this species in [61]). A qualitative shift in behaviour occurred in the T5 treatment; focal fish tended to increase their speed dramatically when neighbours were very near, irrespective of if these neighbours were to the front or rear of the focal individual, and reduce speed in response to neighbours at greater distances, again irrespective of if these neighbours were to the front or the rear (figure 3 *m*). The tendency to turn towards neighbours at intermediate distances persisted in T5 treatments, but the response to very near neighbours observed in the T3 and T4 treatments was reversed in the T5 treatment, with focal individuals turning towards neighbours directly in front, and away from those directly to their rear (figure 3 *n*).

### 3.2. Collective order and states of groups—broad observations

Irrespective of treatment, collective order parameter pairs occurred most frequently for the approximate region in parameter space where $0\;\leq \;O_{r}\;\leq \;0.35$ and $0\;\leq \;O_{p}\;\leq \;0.65$ . The peak frequency for order parameter pairs occurred approximately where $0.15\;\leq \;O_{r}\;\leq \;0.18$ and $0.3\;\leq \;O_{p}\;\leq \;0.35$ , within the region where motion would be automatically classified as swarming (figure 3, rightmost column). Arrows illustrating the mean direction of change of polarization with respect to group angular momentum suggested movement through $\left(O_{r},\;O_{p}\right)$ parameter space towards the peaks within the swarming region across all treatments (figure 3, rightmost column). However, swarming behaviour neither was necessarily stable nor was it the dynamic state most frequently recorded across all observations. From total aggregated count data, groups spent the greatest proportion of their observed behaviour exhibiting behaviour that fell into the composite region of $\left(O_{r},\;O_{p}\right)$ parameter space (54.21–58.01% of the time), followed by swarming (27.37–38.37% of the time), moving in parallel (5.31–13.02% of the time) and milling (1.31–2.58% of the time) (figure 4 *a*).

There were many differences between pairs of treatments in the total proportions of time spent in each collective state. Groups subject to the T3 treatment spent a significantly lower proportion of time milling than T2, T4 and T5 groups (electronic supplementary material, table S3, with boxplots in figure 4 *b*). T1 groups spent a significantly lower proportion of time moving in parallel than T3, T4 and T5 groups (electronic supplementary material, table S4; figure 4 *c*). Similarly, T2 groups spent significantly less time enacting parallel motion than T3, T4 and T5 groups (electronic supplementary material, table S4; figure 4 *c*). T5 groups spent significantly less time in parallel than T3 and T4 groups (electronic supplementary material, table S4; figure 4 *c*). Both T1 and T2 groups spent a significantly greater proportion of time swarming than T3, T4 and T5 groups (electronic supplementary material, table S5; figure 4 *d*). Conversely, T1 groups spent a significantly lower proportion of time enacting composite motion than T3, T4 and T5 groups, and T2 groups spent less time enacting composite motion than T4 and T5 groups (electronic supplementary material, table S6; figure 4 *e*).

### 3.3. Durations of persistence of collective states

**Figure 5.**
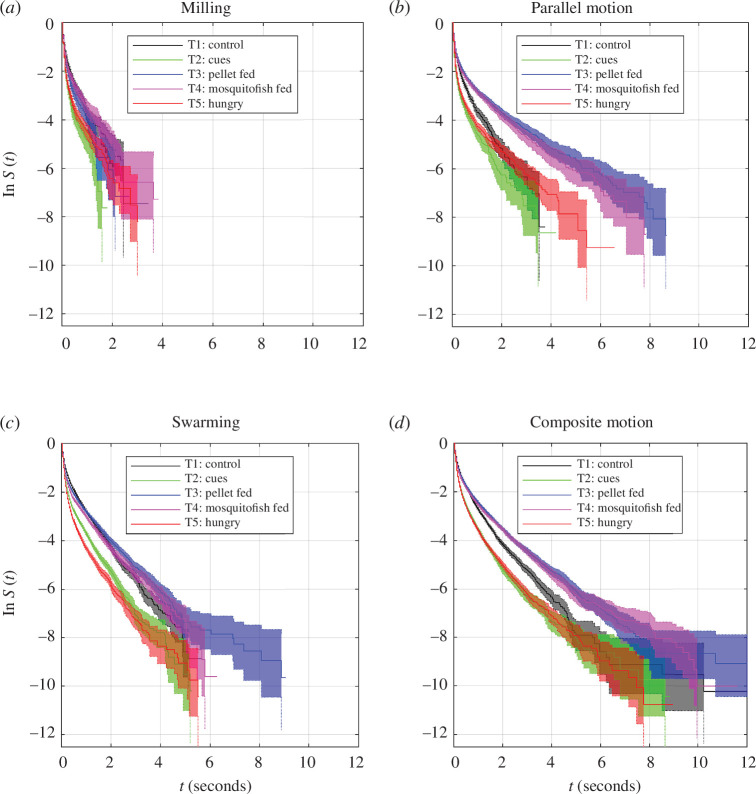
Log-survival curves, ln *S*, for unbroken durations spent by the whole group in each collective state, collated by the form of the collective state ((*a*) milling, (*b*) parallel motion, (*c*) swarming and (*d*) composite motion). 95% confidence intervals for the estimated survival curves are illustrated by shaded regions, bounded by dotted lines. By treatment, the curves correspond to T1 (black curves), T2 (green), T3 (blue), T4 (magenta) and T5 (red).

**Figure 6. F6:**
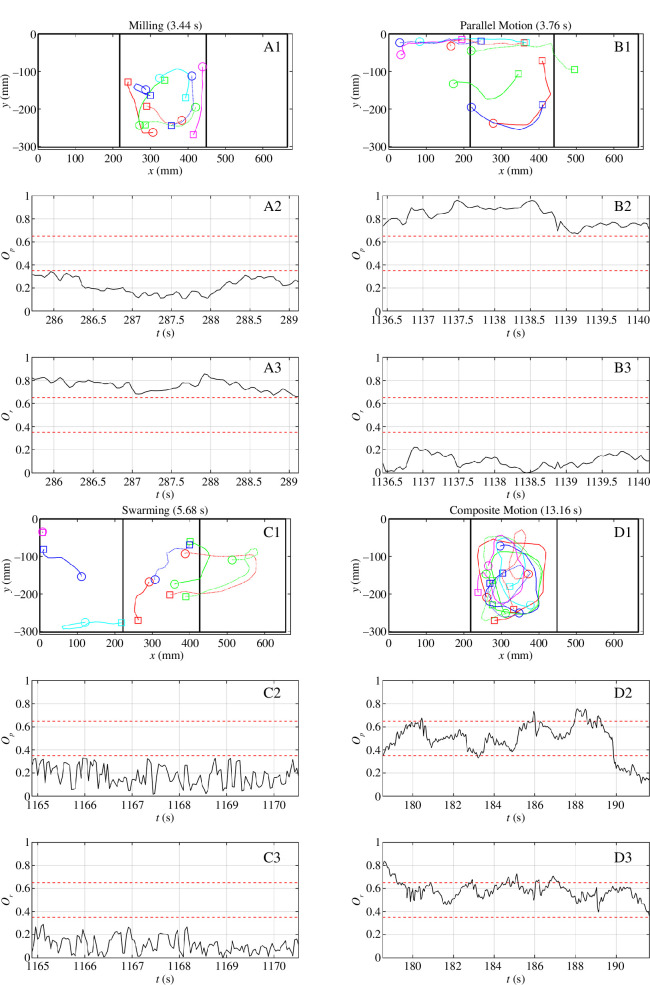
Smoothed individual tracks and associated order parameters for the longest duration instances of milling (A), swarming (B), parallel motion (C) and composite motion (D) in T1 (control). Along each track, each individual’s starting position is marked with a circle, and their position at the end of the group movement is marked with a square. The illustrated tracks were smoothed via a Savitzky–Golay filter (of span 5 and degree 2). Straight black lines on the panels illustrating the tracks represent the boundary of the experimental arena on the exterior, and also demarcate the central chasm region from the platforms to the left and right. $O_{p}$ and $O_{r}$ are the polarization and group angular momentum for the duration that the illustrated groups undertook each form of motion, plotted as a function of time in seconds. Electronic supplementary material, videos S1–S4 provide animations of the movements of the individuals shown in the static images of panels (A1), (B1), (C1) and (D1), respectively.

Durations spent in any particular collective state before transitioning to another state were generally very short, with a very large portion of group patterns of movement changing within 3 s or less (figure 5). The maximum durations observed in the milling, parallel, swarming and composite states were 3.84 s (within the T4 (mosquitofish-fed predator) observations), 8.68 s (T3 (pellet-fed predator)), 9.08 s (T3 (pellet-fed predator) and 13.16 s (T1 (control)) across all treatments. To gain a better understanding of what the group motion actually looked like during the brief periods spent in each form of collective state, we extracted the longest duration instances of milling, parallel motion, swarming and composite motion from the T1 (control) data, along with the corresponding time series of the polarization and group angular momentum order parameters. During the brief instance of exemplar milling behaviour (of duration 3.44 s), the bulk of the group of eight fish rotated in a largely consistent clockwise sense over the central chasm region of the tank (figure 6A1; electronic supplementary material, video S1). The instance identified as parallel motion (3.76 s) did not entirely comprise individuals following straight paths, and involved individuals moving from on or near the left platform towards the right platform region (figure 6; electronic supplementary material, video S2). The longest instance of swarming motion (5.68 s) involved motion that did not seem to involve whole-group coordination (figure 6C1; electronic supplementary material, video S3). On the other hand, the longest instance of composite motion (13.16 s, and hence the longest duration of this form of motion across any treatment) from the T1 data set involved coherent motion of the group moving around the central chasm region in a clockwise sense (figure 6D1; electronic supplementary material, video S4).

### 3.4. Differences in durations of collective states across treatments

The persistence of collective patterns of movement was affected by indicators of predation risk. Most pairs of treatments differed significantly in the durations spent in milling, parallel, swarming and composite states, even if these differences were small in practice (electronic supplementary material, tables S7–S10; figure 5). There were significant differences for durations spent milling between T1 and T2, T1 and T3, T1 and T5, T2 and T3, T2 and T4, T3 and T4, T3 and T5, and T4 and T5 treatments (electronic supplementary material, table S7). Of these, T2 and T5 groups tended to spend shorter unbroken durations milling than T1, T3 and T4 treatments (figure 5 *a*). All treatments differed in the unbroken durations engaged in parallel motion (electronic supplementary material, table S8); typically, T2 and T5 groups spent shorter durations in parallel motion than the other treatments (figure 5 *b*). Likewise, all treatments differed in durations in a swarming state (electronic supplementary material, table S9), with T2 and T5 groups maintaining swarm-like motion for the shortest durations (figure 5 *c*). T1 and T2, T1 and T3, T1 and T5, T2 and T3, T2 and T4, T3 and T4, T3 and T5, and T4 and T5 treatments all differed in durations spent in composite motion (electronic supplementary material, table S10). Groups in the T2 and T5 treatments tended to spend less unbroken time in composite motion than those in T1, T3 and T4 (figure 5 *d*).

### 3.5. Differences between durations of collective states within treatments

**Figure 7.**
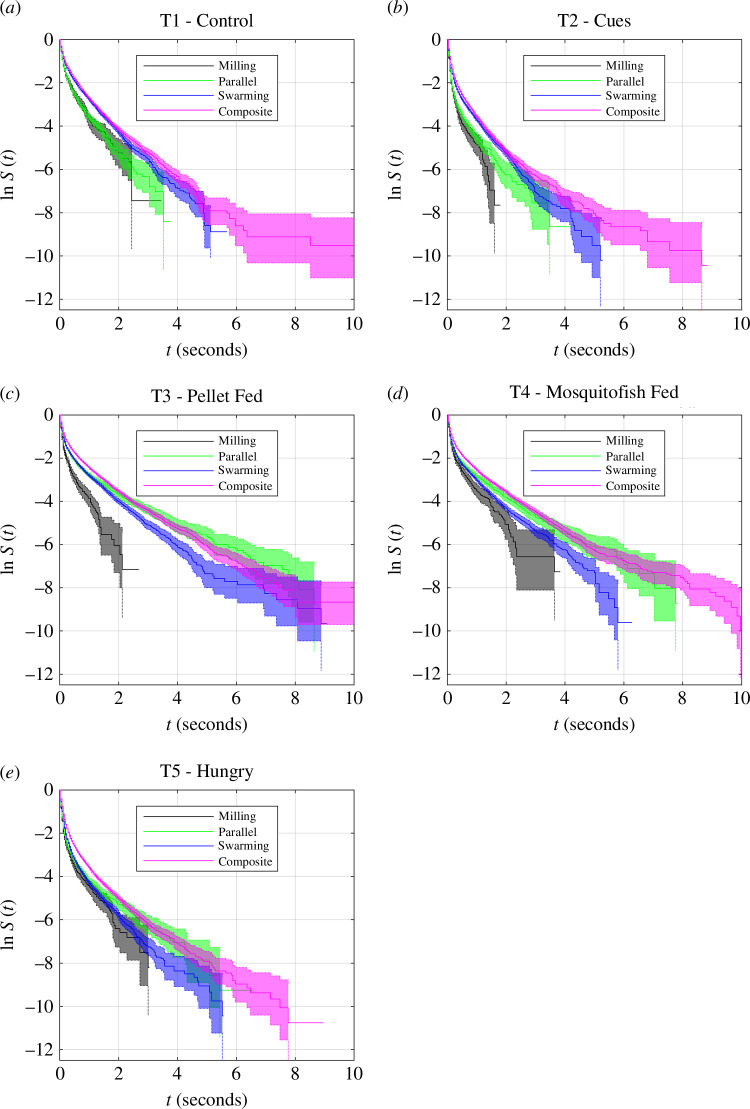
Log-survival curves, ln *S*, for unbroken durations spent by the whole group in each collective state, collated within the treatment (*a*) T1 (control), (*b*) T2 (cues), (*c*) T3 (pellet fed), (*d*) T4 (mosquitofish fed), (e) T5 (hungry predator). 95% confidence intervals for the estimated survival curves are illustrated by shaded regions, bounded by dotted lines. By state, the curves correspond to milling (black curves), parallel motion (green), swarming (blue) and composite motion (magenta).

For all treatments, composite motion tended to persist for the longest durations, again noting the very short durations for the persistence of any collective state. In the T1 and T2 treatments, where the central zone was unoccupied, durations of milling and moving in parallel tended to last the least (figure 7 *a*,*b*). For treatments where the predator occupied the central zone (T3, T4 and T5) milling was the single least persistent state, with parallel motion ultimately lasting longer than swarming (figure 7 *c*–*e*). In detail, within the T1 treatment, durations spent in all pairings of collective states differed, except for milling and parallel motion (electronic supplementary material, table S11). Milling and parallel motion persisted for the shortest durations, with survival curves that match each other closely (even on a logarithmic scale), followed by swarming, with composite motion tending to persist the longest (figure 7 *a*). In the T2 treatment, all pairings of the collective state differed in duration (electronic supplementary material, table S12). As with the T1 treatment, milling and parallel motion persisted for the shortest durations in T2 groups, followed by parallel motion, and greater durations of persistence in composite motion (figure 7 *b*). All pairings of collective state differed in duration, except parallel motion and swarming, for both the T3 and T4 treatments (electronic supplementary material, tables S13 and S14). In T3 and T4, milling clearly persisted for the shortest durations (figure 7 *c*,*d*). For the T5 treatment, all pairings of collective states differed in their durations of persistence (electronic supplementary material, table S15), with composite motion being the most persistent (figure 7 *e*).

### 3.6. Associations between interaction rules and persistence of collective states

Given that there were at least some significant differences between all pairs of treatments in the durations that collective states persisted, differences in these details of the collective states coincided with differences in force-mapping inferred interaction rules (table 1). However, in the case of comparisons between the T1 (control) and T2 (predator cues), and T3 (pellet-fed predator) and T4 (mosquitofish-fed predator) treatments, differences in the durations of persistence of specific collective states were also identified when there was no significant difference in inferred interaction rules (table 1). Inspection of the survival curves in figure 5 suggests that although differences between the T3 and T4 treatments were significant, in practice these differences were relatively small, since the curves match each other quite closely for each collective state (compare the blue and magenta curves and their 95% confidence intervals in figure 5). On the other hand, the durations of persistence of collective states of the T1 and T2 treatments are distinct from each other, especially for shorter durations (figure 5, black and green curves and associated 95% confidence intervals).

**Table 1.** Summary of significant differences identified in force-map inferred changes in speed and direction, and unbroken durations in collective state, between all pairs of treatments (derived from data in electronic supplementary material, tables S1, S2 and S7–S10). Below. a **‘**✓' indicates that a statistically significant difference was identified. The treatments were: T1—control; T2—predator cues from predator in separate outermost region; T3—predator fed on pellets and present in central region; T4—predator fed on mosquitofish and present in central region; and T5—predator hungry and present in central region.

	Δs/Δt	Δθ/Δt	mill	parallel	swarm	composite
**p**air of treatments						
T1, T2			✓	✓	✓	✓
T1, T3	✓		✓	✓	✓	✓
T1, T4	✓	✓		✓	✓	
T1, T5	✓	✓	✓	✓	✓	✓
T2, T3	✓		✓	✓	✓	✓
T2, T4	✓		✓	✓	✓	✓
T2, T5		✓		✓	✓	
T3, T4			✓	✓	✓	✓
T3, T5	✓	✓	✓	✓	✓	✓
T4, T5		✓	✓	✓	✓	✓

### 3.7. Transitions between collective states

**Figure 8.**
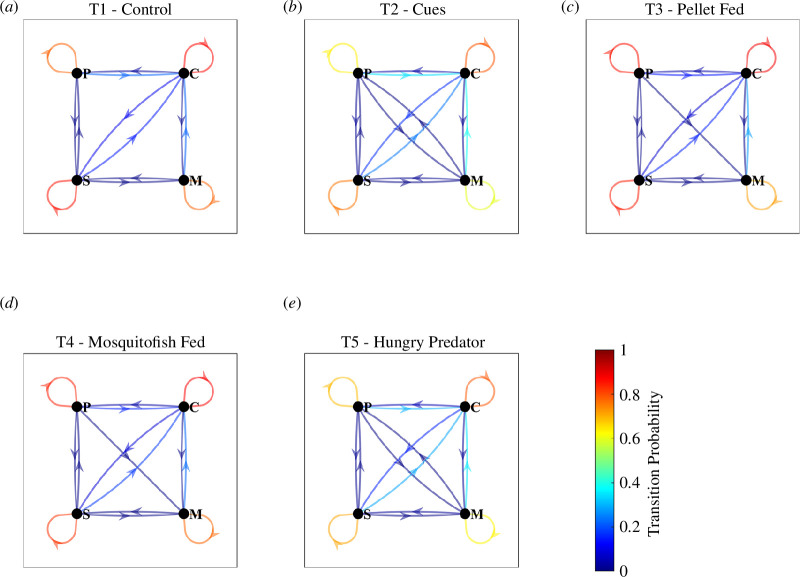
Observed relative frequencies (probabilities) of transitions between collective states at each video frame/discrete time step of data. Arrows indicate the direction of each transition, loops indicate no change in state, and the colour scale indicates the transition probability, ranging from 0 (dark blue) to 1 (dark red).

Treatment, and thus indicators of predation risk, had an effect on the relative frequency of direct transition between collective states (figure 8). In all treatments, the probabilities of remaining in any current collective state were greater than that of transitioning to another state. Direct transitions in both directions between all pairs of collective states were observed for the T2 and T5 treatments, with similar transition probabilities between all pairs of states. T3 and T4 treatments exhibited very similar transition probability patterns as well, with direct transitions in both directions between all states, except from milling to parallel motion. The T1 treatment exhibited transitions between most states, except shifts from parallel motion to milling, and from milling to parallel motion (figure 8 *a*).

### 3.8. Formation, persistence and average structure of subgroups

#### 3.8.1. Subgroups of different sizes within treatments

**Figure 9.**
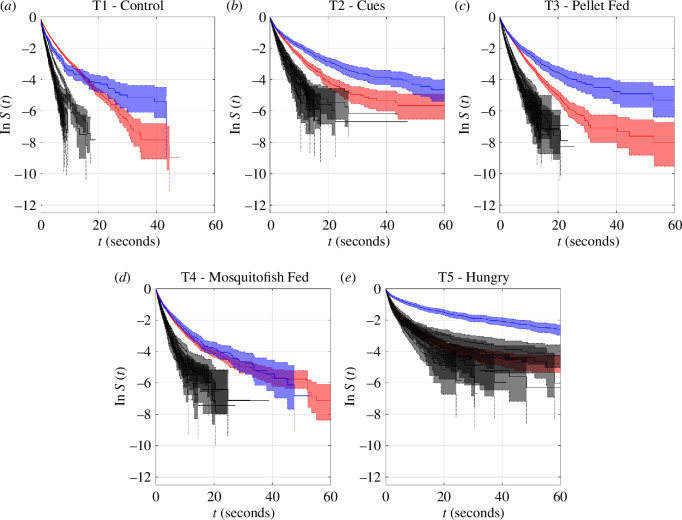
Log-survival functions of durations that subgroups of different sizes persisted within each treatment, with 95% confidence intervals (shaded regions), ((*a*) T1 (control), (*b*) T2 (cues), (*c*) T3 (pellet fed), (*d*) T4 (mosquitofish fed), (*e*) T5 (hungry predator)). Survival functions for subgroups of 1 are plotted in red, and survival functions for subgroups of size 8 are plotted in blue. Survival curves for all other subgroup sizes are plotted in black. The criteria for subgroup membership was based on subgroup members being within four body lengths of each other.

The durations that subgroups of different sizes persisted differed between at least one pair of subgroup sizes in all treatments (log-rank test (d.f. = 7 for all tests): control (T1), *p* ≈ 0, test-statistic ≈ 2837; predator cues (T2), *p* ≈ 0, test-statistic ≈ 616.3; predator fed with pellets (T3), *p* ≈ 0, test-statistic ≈ 1794; predator fed with mosquitofish (T4), *p* ≈ 0, test-statistic ≈ 930.6; hungry predator (T5), *p* ≈ 0, test-statistic ≈ 329.0). Many pairs of subgroups differed in their empirical survival functions for durations of persistence (see electronic supplementary material, tables S17–S20). The number of differences ranged from 14 out of 28 pairings of subgroup sizes differing in the hungry predator treatment (T5) to 24 out of 28 pairings differing in the control treatment (T1). Subgroups of size 8 differed in their durations of persistence compared with all other subgroups in T1, T2, T3 and T5. Subgroups of size 1 differed in their duration of persistence compared with all other subgroups for treatments T1 through to T3; subgroups of size 2 differed from all other groups in T1, T3 and T4, and subgroups of size 3 differed from all other groups in T1. Examination of the empirical survival functions in figure 9 reveals that subgroups of size 8 tended to persist for longer than all other groups in treatments T2, T3 and T5, whereas in T1 and T4 the subgroup of 8 survival curve crosses over with the subgroup of 1 survival curve. For T2 and T3, subgroups of 1 tended to persist for the second longest duration, but under the greatest perceivable risk of predation in T5, subgroups of 1 persisted for similar durations to that of other subgroups of 7 or less (again, see figure 9; there were no significant differences between survival curves for subgroups of 1 and 3, 1 and 4, and 1 and 7 in T5 according to our log-rank tests; electronic supplementary material, table S20).

### 3.8.2. Subgroups of fixed sizes across treatments

**Figure 10.**
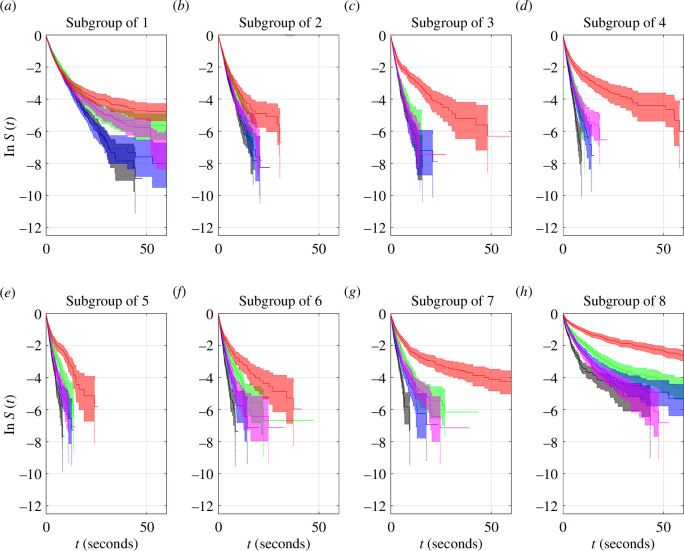
Log-survival functions of durations that subgroups of different sizes persisted across treatments, with 95% confidence intervals (shaded regions). The survival curves are coloured as follows: T1 (control—black), T2 (predator cue—green), T3 (predator fed with pellets—blue), T4 (predator fed with mosquitofish—magenta) and T5 (hungry predator—red). Subgroup sizes range from 1 (*a*) to 8 (*h*)

For fixed subgroup sizes, durations of subgroup persistence differed between at least one pair of treatments (log-rank test (d.f. = 4 for all tests): singletons, *p* = 5.42 × 10^−7^, test-statistic ≈ 34.68; subgroups of 2, *p* = 1.32 × 10^−12^, test-statistic ≈ 61.63 ; subgroups of 3, *p* ≈ 0, test-statistic ≈ 145.3; subgroups of 4, *p* ≈ 0, test-statistic ≈ 198.6; subgroups of 5, *p* ≈ 0, test-statistic ≈ 176.2; subgroups of 6, *p* ≈ 0, test-statistic ≈ 192.5; subgroups of 7, *p* ≈ 0, test-statistic ≈ 339.2; subgroups of 8, *p* ≈ 0, test-statistic ≈ 409.0). Subsequent pairwise comparisons showed that subgroup durations differed across the majority of pairings of treatments, except for subgroups of size 1 (see electronic supplementary material, tables S21–S28). Subgroups of 1, 2, 3, 5 and 8 persisted for similar durations within the T3 and T4 treatments. Durations of subgroup persistence did not differ between: T1 and T4 for subgroups of 1; T1 and T5 for subgroups of 1; T3 and T5 for subgroups of 1; T1 and T3 for subgroups of 1 and 3; T4 and T5 for subgroups of 1; T2 and T5 for subgroups of 2, 5 and 6; T2 and T3 for subgroups of 3; and T2 and T4 for subgroups of 3 and 4. Examination of the Kaplan–Meier estimates of the empirical survival functions shows that subgroups of all sizes tended to persist longer for T5 than under any other treatment (figure 10; note, however that log-rank tests suggested no significant difference between T2 and T5 survival curves for subgroups of 5 and 6 as indicated in electronic supplementary material, tables S25 and S26).

Mean median subgroup size was not normally distributed for T1 (control), T2 (predator cues) and T5 (hungry predator) (control (T1)—Shapiro–Francia test, p = 0.0056 , W′ = 0.7846 ; predator cues (T2)—Shapiro–Wilk test, p = 0.0315 , W = 0.8278 ; predator fed with pellets (T3)—Shapiro–Wilk test, p = 0.8500 , W = 0.9648 ; predator fed with mosquitofish (T4)—Shapiro–Wilk test, p = 0.6862 , W = 0.9533 ; hungry predator (T5)—Shapiro–Francia test, p = 0.0019 , W′ = 0.7347 ). Visual inspection of histograms of mean median subgroup size suggested that the distributions of this measure clearly differed in shape across treatments (figure *2b–f*). Pairwise comparisons with two-sample Kolmogorov–Smirnov tests then showed that mean ranks differed between T1 and T2, T1 and T5, and T1 and T4 (electronic supplementary material, table S29). Fish in control (T1) groups tended to have lower mean median subgroup sizes, whereas the largest mean median subgroup sizes were associated with predator cue (T2) and hungry predator (T5) treatments (figure *2a*).

#### 3.8.3. Local density, alignment and collective order of subgroups

**Figure 11.**
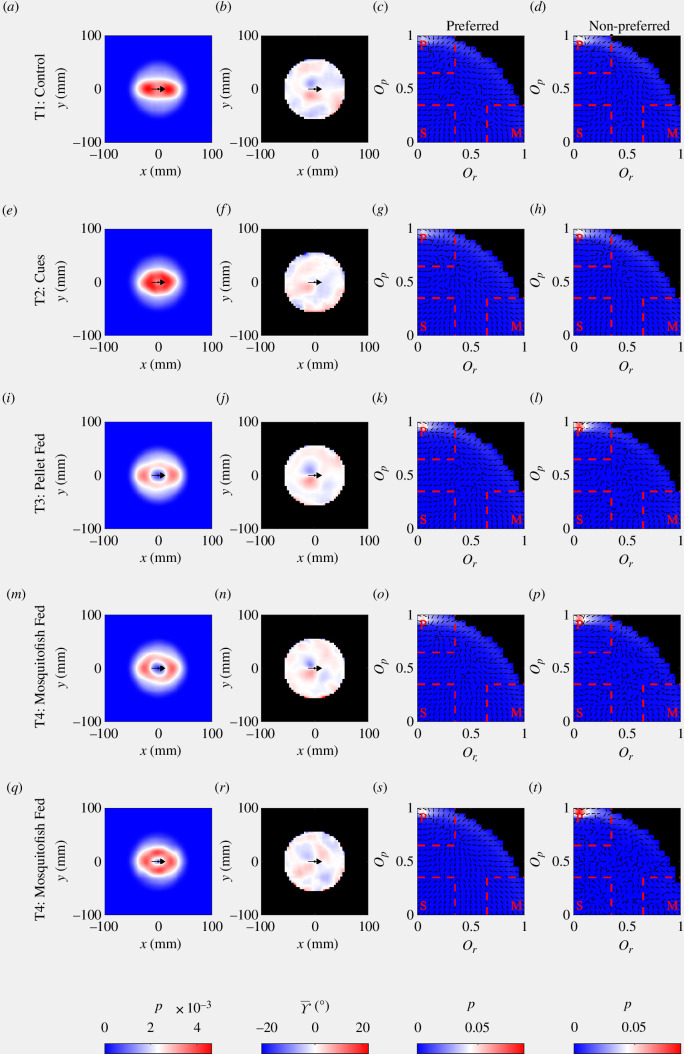
Local subgroup structure, alignment and collective order parameter summary for subgroups of 2. Each row corresponds to a different treatment, as indicated by the leftmost labels. The leftmost column (*a*,*e*,*i*,*m*,*q*) illustrates the relative frequency that mosquitofish occupied particular positions relative to the subgroup centroid, and the second column from the left (*b*,*f*,*j*,*n*,*r*) illustrates the direction of motion of subgroup members relative to that of the subgroup centroid, as a function of the relative positions of the fish. In each plot in the left two columns, the group centre is located at (0, 0), such that the direction of motion of the subgroup centre is parallel to the positive *x*-axis (the position and heading of the group centre is represented by small black arrows in the left two columns). In the relative alignment plots, blue colours indicate that individuals tended to be rotated clockwise relative to the positive *x*-axis and red colours indicate that individuals tended to be rotated anticlockwise relative to the positive *x*-axis. Note that the colour scale on the relative alignment plots has been truncated at ±20° to clarify the sense (clockwise or anticlockwise) of relatively small angular differences. The right two columns illustrate the relative frequency that particular order parameter pairs $\left(O_{r},\;O_{p}\right)$ , occurred across all observations for a given treatment, in preferred (third column from the left, (*c*,*g*,*k*,*o*,*s*)) and non-preferred (fourth column from the left, (*d*,*h*,*l*,*p*,*t*)) regions of the experimental aquaria; arrows in these plots indicate the average direction of $dO_{p}/dO_{r}$ . For T1 and T2, the preferred zone (determined by the total time spent in each zone) was the central zone, whereas for T3, T4 and T5, the preferred zones were the platform regions. Conversely, the non-preferred zones were the platform regions for T1 and T2 groups, and the central zone for T3, T4 and T5.

**Figure 12.**
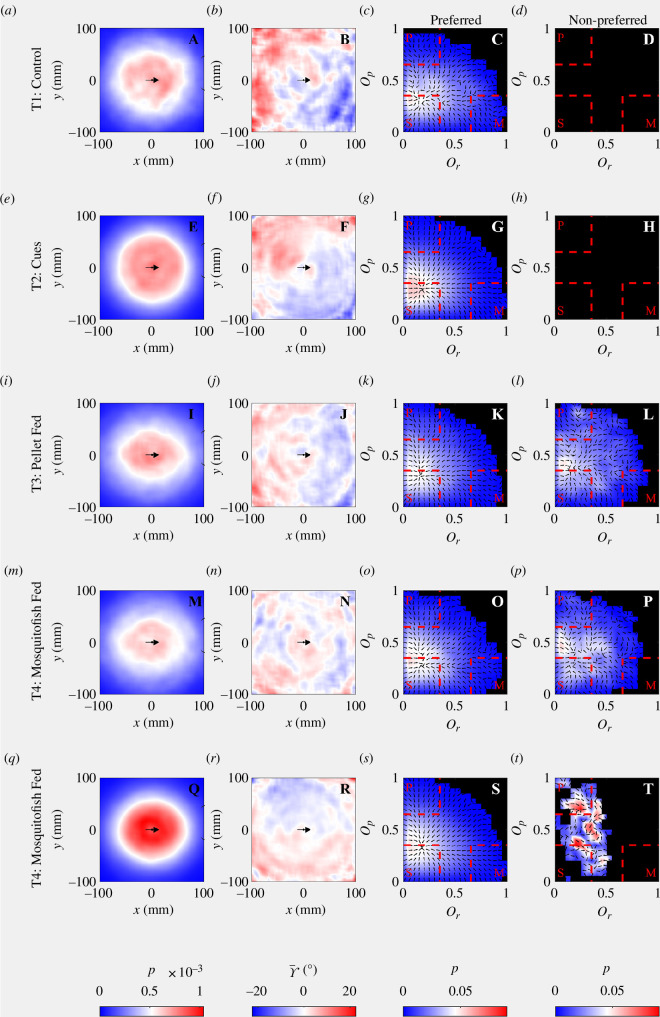
Local subgroup structure, alignment and collective order parameter summary for subgroups of 8 (i.e. the entire group when all group members were relatively close to each other). The leftmost column (*a*,*e*,*i*,*m*,*q*) illustrates the relative frequency that mosquitofish occupied particular positions relative to the subgroup centroid, and the second column from the left (*b*,*f*,*j*,*n*,*r*) illustrates the direction of motion of subgroup members relative to that of the subgroup centroid, as a function of the relative positions of the fish. In each plot in the left two columns, the group centre is located at (0, 0), such that the direction of motion of the subgroup centre is parallel to the positive *x*-axis (the position and heading of the group centre is represented by small black arrows in the left two columns). In the relative alignment plots, blue colours indicate that individuals tended to be rotated clockwise relative to the positive *x*-axis and red colours indicate that individuals tended to be rotated anticlockwise relative to the positive *x*-axis. Note that the colour scale on the relative alignment plots has been truncated at ±20° to clarify the sense (clockwise or anticlockwise) of relatively small angular differences. The right two columns illustrate the relative frequency that particular order parameter pairs $\left(O_{r},\;O_{p}\right)$ , occurred across all observations for a given treatment, in preferred (third column from the left, (*c*,*g*,*k*,*o*,*s*)) and non-preferred (fourth column from the left, (*d*,*h*,*l*,*p*,*t*)) regions of the experimental aquaria; arrows in these plots indicate the average direction of $dO_{p}/dO_{r}$ . For T1 and T2, the preferred zone (determined by the total time spent in each zone) was the central zone, whereas for T3, T4 and T5, the preferred zones were the platform regions. Conversely, the non-preferred zones were the platform regions for T1 and T2 groups, and the central zone for T3, T4 and T5.

Patterns in plots of the relative frequency that mosquitofish occupied particular (x, y) coordinates relative to the location and direction of motion of the subgroup centroid for subgroups of 2 were consistent with fish often moving in single file in T1 to T4 (figure 11*a,e*,*i*,*m*), but this pattern is broken in T5 where partners often adopted side-by-side configurations as well as single file configurations (figure 11*q*). Across subgroups from size 3 to 8, subgroup members tended to be quite diffuse in T3 and T4, and perhaps most densely packed (evidenced by greater relative frequencies) in T5 (electronic supplementary material, figures S1–S5; figure 12, left column). Mean angular differences between the direction of motion of individuals and that of the subgroup centroid tended to be relatively small (figures 11 and 12; electronic supplementary material, figures S1–S5*b*,*f*,*j*,*n*,*r*). Within subgroups of 2, individuals tended to move towards the centre of the pair when they were behind, but close to the centroid, and fan out, away from the centre when slightly in front of this position, across all treatments (figure 11); the magnitudes of the associated angular differences were smaller in the T2 and T5 treatments than in the T1, T3 and T4 treatments. General patterns in the sense (clockwise or anticlockwise) of angular differences were not readily apparent for subgroups of 3–8 (electronic supplementary material, figures S1–S5; figure 12*b*,*f*,*j*,*n*,*r*).

Subgroups of 2 and 3 most frequently engaged in parallel motion, irrespective of their occupancy of their preferred or non-preferred zone and treatment (figure 11 and electronic supplementary material, figure S1, rightmost columns). The most frequent pairings of order parameters and associated forms of collective motion were affected by treatment for subgroups of 4 or more. In T1, subgroups of 4 most frequently engaged in composite motion, with $0\;\leq \;O_{r}\;\leq \;0.5$ and $0.35<O_{p}<0.65$ , irrespective of occupancy of their preferred or non-preferred zone (the central zone or the platforms, respectively; electronic supplementary material, figure S2, rightmost columns). Subgroups of 4 in T2 most frequently engaged in composite motion in their preferred zone (the central zone), similar to T1, and engaged in both composite and parallel motion with similar relative frequencies in their non-preferred zone (the platforms). Subgroups of 4 were most frequently engaged in highly parallel motion in T3 and T4, with $O_{p}$ close to 1, with this tendency strongest when the fish were in their non-preferred zone (the central zone for these treatments, occupied by the satiated predator (perch)). T3 and T4 subgroups of 4, also engaged in composite motion, with $0\;\leq \;O_{r}\;\leq \;0.5$ and $0.35<O_{p}<0.65$ (approximately), at relatively high frequency. The movements of subgroups of 4 in T5 resulted in similar patterns in distributions of collective order parameters to T3 and T4 in their preferred zone (the platforms), and rarely engaged in milling behaviour (electronic supplementary material, figure S2). In their preferred zone, subgroups of 5 in T1 most frequently engaged in motion where $0<O_{r}<0.35$ and $0<O_{p}<0.65$ , encompassing both swarm-like and composite behaviour (electronic supplementary material, figure S3). Similar patterns were evident for T2 and T4, whereas T2 and T5 subgroups of 5 also engaged in parallel motion with relatively high frequency. Patterns in order parameter space differed markedly across treatments for subgroups of 5 (electronic supplementary material, figure S3). As subgroup size increased from 6 to 8, the patterns in order parameter space for all treatments in the preferred zone, and T3 and T4 in their non-preferred zone changed to resemble that for the whole group (without any distance-based threshold for subgroup membership) illustrated in figure 3, with swarming and composite motion most frequent (electronic supplementary material, figures S4 and S5; figure 12, rightmost columns). Milling was observed infrequently, if it all, when subgroups of 6–8 occupied their non-preferred zone. Highly parallel motion was relatively frequent for subgroups of 6–8 traversing their non-preferred zone in T5 (electronic supplementary material, figures S4 and S5; figure 12).

### 3.9. Patterns of differences across and within treatments

We tabulated the results of all inferential statistical tests applied between pairs of treatments for all 19 measures to which these tests were applied, including changes in speed and changes in direction previously reported in Wilson *et al*. [47] (see electronic supplementary material, table S30). In addition, we tabulated results relating to comparisons of the durations of unbroken bouts of milling, parallel motion, swarming and composite motion, and the durations that subgroups of different sizes persisted within each treatment (see electronic supplementary material, tables S31 and S32). For the within-treatments comparisons, we compared the tabulated results across treatments, to identify if the patterns of differences, or lack thereof, were similar between given pairs of treatments.

Treatments where the predator (perch) was present in the central zone, and had been fed pellets (T3) or dead mosquitofish (T4) had the fewest statistical differences (8 out of 19) across the raft of measures examined (electronic supplementary material, table S30). The differences identified between T3 and T4 were in the total proportion of time spent milling, the durations of unbroken bouts of milling, parallel motion, swarming and composite motion, and the durations that subgroups of 4, 6 and 7 persisted. The next fewest statistical differences were for comparisons between T2 (the predator cues treatment) and T5 (where the predator (perch) was not fed before being placed in the central zone), with a total of 12 out of 19 differences (electronic supplementary material, table S30). The most differences occurred in comparisons of T1 (control) and T5, with 17 measures identified as differing between the treatments except for the total proportion of time spent milling, and the duration that subgroups of 1 persisted (i.e. the duration that individuals were spatially separated from other members of the group/shoal).

In terms of the presence or lack of significant differences in the durations that particular states endured within each treatment, the most similar pairs of treatments were T2 and T5, where the durations of persistence of all states differed with all other states (identical results for all pairs of states), and T3 and T4, where all pairs of states differed in duration, except parallel motion and swarming (again with identical results for all pairs of states) (electronic supplementary material, table S31). There were 28 pairwise comparisons for the durations of persistence of subgroups of different sizes within each treatment. Of these, T3 and T4 had the same results in 25 cases, with the only differences being for subgroups of 1 and 8, where a significant difference in survival curves was identified in T3, but not T4; subgroups of 3 and 6 (significantly different in T3, but not T4); and subgroups of 4 and 5 (significantly different in T4, but not T3) (electronic supplementary material, table S32). T1 and T5 were the least similar, with the same results in 14 comparisons.

## 4. Discussion

The hunger state and location of the predator (jade perch) affected many measures of the collective states of the small shoals of mosquitofish, including the overall proportions of time spent in each state, the unbroken durations spent in each state (noting that the duration of persistence of any state was short, 13.16 s or less in our observations), the probabilities of transitions between states, and the most frequent $\left(O_{r},\;O_{p}\right)$ pairs for subgroups. In addition, median subgroup size, the durations that subgroups of different sizes persisted, and the statistical structure of subgroups were all affected by treatment. As noted in §1, previous work on this same experimental system has documented differences in platform crossing behaviour, speed, neighbour distances and density, polarization and local alignment, the predictability of position and velocity changes, and interaction rules (as reiterated in this paper) due to treatment [47]. Combined, these observations suggest that mosquitofish do employ context-sensitive behavioural adjustments in different threat scenarios, rather than just switching to a single form of threat-mediated behaviour irrespective of the form or level of threat, and that these behavioural adjustments apply at the levels of individuals, their interactions and the collective behaviour of the group.

In general, differences between treatments were nuanced, with no two treatments being completely alike in behavioural adjustments across all the measures examined here. The two treatments that seemed to differ the least were T3 (pellet-fed predator (perch)) and T4 (mosquitofish-fed predator (perch)), both of which included the predator (perch), fed, in the central zone of the experimental tank. Prior work on this system also suggested that T3 and T4 did not differ in the overall time spent by mosquitofish on the platform regions (the preferred zone for fish in both these treatments), the statistical density of near neighbours and the relative alignment with these neighbours (with focal individuals used as a point of reference), nor in adjustments to speed or heading in response to the positions of neighbours (as re-examined here) [47]. There was also a degree of similarity between T2 (predator cues) and T5 (hungry predator (perch)), treatments in which the presence of the predator was apparent through either chemical or visual cues. T2 and T5 had the second lowest number of statistical differences for the measures examined here, and also had the same number of statistical differences in durations spent in each collective state, albeit with different states persisting for different relative durations within each treatment (compare panels *b* and *e* of figure 7). Some past studies suggest that some species require both chemical and visual cues to elicit a response to a predator [62,63], whereas here the presence of chemical cues alone were enough to elicit a response (T2), and that response was similar, but not identical, in some respects to the presence of a hungry predator (T5, where visual and chemical cues are both present). The two treatments that differed the most frequently were T1 (control) and T5 (hungry predator (perch)), which represented the opposite extremes of danger to the mosquitofish in the system studied, from the otherwise empty aquaria of the control trials to the presence of a hungry predator (perch). T5 is also the treatment for which the most dramatic qualitative difference in interaction rules was observed.

Prior work, noted in §1, has examined adjustments in grouping behaviour in the presence of chemical cues designed to simulate recent predation of conspecifics, most similar to T2 (predator cues) here, where the predator’s cues mixed freely with the area accessible by the mosquitofish. The tendency for fish to group more closely in the presence of cues, or more imminent threat, has now been demonstrated for multiple species. Hoare et al. [46] observed that for banded killifish, group size, equivalent to subgroup size here, increased in the presence of alarm cues. We observed similar results here, with clearly larger mean median subgroup sizes compared with the control case occurring in the presence of predator cues (T2) or the hungry predator (T5), and to a lesser extent when the predator had been fed dead mosquitofish (T4). Equivalently, Schaerf *et al*. [36] observed decreases in the spacing of individuals in groups of X-ray tetras in the presence of alarm cues. Prior work on the system studied here indicated reductions in nearest neighbour distances in the T2 and T5 treatments when fish occupied their preferred zone (the central zone in T2, and the platforms in T5) [47]. In concert with the tendency to occupy subgroups of larger sizes, there was another aspect to the grouping behaviour of fish in T5; fish in this treatment tended to form subgroups of all possible sizes that persisted for longer durations than in other treatments, that is, the mosquitofish in T5 tended to change subgroups less frequently, or maintained consistent subgroup membership for longer durations. In part, this is consistent with the fish tending to move at slower speed overall when in their preferred regions of the tank—the platforms where they also spent the vast majority of their time [47].

Mosquitofish in the presence of the predator (perch) (T3–T5) spent a greater proportion of time engaged in parallel motion than in treatments where the predator was absent from the central portion of the arena. Subgroups of fish in T3–T5 also moved in parallel at relatively high frequency when in the non-preferred central zone. This may have been because of the necessary alignment between individuals crossing in a group from platform to platform across the central zone, or perhaps illustrated a tendency to engage in more orderly behaviour (at the group level) when in the presence of an apparent threat. It is not always the case that fish in general become more highly aligned in the presence of potential threat though. For example, X-ray tetras have been shown to become less aligned with other group members when subject to alarm cues [36].

When viewed via aggregated data for the whole group, the relative frequency that particular forms of order appeared did not seem to vary much qualitatively between treatments. The regions in order parameter space most frequently occupied corresponded to swarming behaviour, and composite behaviour sitting between the swarming and parallel motion regions. However, overall proportions of time spent in distinct collective states did differ between treatments, and all pairs of treatments differed in unbroken durations spent in a particular state for at least two of the four states classified here. Given that in previous work differences between most pairs of treatments were identified for at least one of the components of changes in velocity as a function of neighbour location, there is an association between differing rules of interaction and the details of emergent group-level patterns of motion for mosquitofish. The exceptional cases were comparisons of T1 and T2, and T3 and T4, where there were no differences detected in interaction rules, but where there were differences in unbroken durations spent in each collective state. Theoretically, it is possible for different collective states to emerge from the same underlying rules of interaction, as models for collective motion can exhibit what appears to be initial condition dependence [41,64] and hysteresis [23,41].

Theoretical models often generate single and relatively stable long-term collective states, including models shown to exhibit initial condition and system history dependence, whereas the behaviour of the mosquitofish studied here is much more complex and dynamic, with no long-term occupancy of any clear collective state. Similar short-term occupancy of collective states has also been observed in the context of the collective order of competitive football and rugby sevens teams, during both attacking and defending phases of play [40,65]. Thus, rapid dynamic changes in collective order are a quantifiable characteristic of collective motion across taxa and context, in some instances. Longer duration maintenance of collective states seems to be characteristic of larger group sizes. Prior studies of larger groups of fish, specifically golden shiners (*Notemigonus crysoleucas*) in Tunstrøm *et al*. [39] and rummy-nose tetras (*Hemmigrammus rhodostomus*) in Lafoux *et al*. [66], illustrate that fish shoals are capable of maintaining distinct patterns of collective movement over periods of many minutes in laboratory aquaria, with instances of milling persisting for greater durations as group size increases [39]. Transitions in collective state for golden shiner shoals were shown to be instigated spontaneously by either group members or via interactions with boundaries in [39], whereas emergent state was affected by ambient light intensity and the aquaria boundary for rummy-nose tetras [66]. In addition to predation threat, the geometry of our experimental aquaria probably affected the paths taken by the mosquitofish studied here as well, for example, the instance of milling illustrated in [Fig F6] figure 6A1 and electronic supplementary material, video S1 lay entirely within the central chasm region, and the instance of parallel motion was connected with movement from one platform region to another (figure 6B1; electronic supplementary material, video S2).

The rules of interaction examined here and in Wilson *et al*. [47] were inferred via force mapping, an approach that has a reasonable degree of reliability in identifying the qualitative, and sometimes the quantitative, form of interaction rules [64]. Force mapping also has inaccuracies relating to interactions combined in response to multiple individuals [64], interaction rules that apply and are combined over the same spatial range [32], and long-term emergent patterns of motion [64]. Given that the analysis presented here has identified the qualitative form of repulsion and attraction interactions under a number of potential threat scenarios, including the markedly different behaviour observed under T5 (which could not have been easily anticipated without force mapping), a next step in improving the analysis presented here could be to fit appropriately chosen functions to the data using the approach developed across [28–30,32]. These fitted functions could then form the basis for data-driven models for collective motion in the framework described by Escobedo *et al*. [32]. The behaviour of the models for each treatment could then be validated against experimental data using a number of individual and collective measures, including measures of subgroup formation and collective order like those used in this study, in the process establishing a more causal relationship between interactions and collective movement patterns. Such future work then has the potential to better resolve the relationship between interactions and differences in elements of collective movement across the contexts examined here.

At the group level, the eastern mosquitofish examined here demonstrated different behavioural profiles dependent on threat scenario treatment. It will be of interest in future studies to examine the behaviour of other species over similar graded ecological conditions, to better understand if what has been observed here and in Wilson *et al*. [47] might represent a broad principle, or is specific to some species, but not others.

## Data Availability

All data and custom analysis codes (written in MATLAB) are available as part of the electronic supplementary material [67].
